# Neonatal factors related to center variation in the incidence of late-onset circulatory collapse in extremely preterm infants

**DOI:** 10.1371/journal.pone.0198518

**Published:** 2018-06-12

**Authors:** Yume Suzuki, Yumi Kono, Takahiro Hayakawa, Hironori Shimozawa, Miyuki Matano, Yukari Yada, for the Neonatal Research Network Japan

**Affiliations:** 1 Department of Pediatrics, Jichi Medical University, Shimotsuke city, Tochigi Pref., Japan; 2 Medical Policy Division, Department of Health and Welfare Services, Tochigi Prefectural Office, Utsunomiya city, Tochigi Pref., Japan; Massachusetts Institute of Technology, UNITED STATES

## Abstract

**Background:**

Although late-onset circulatory collapse (LCC) is widely recognized in Japan, its etiology and the reason for center variation in its incidence remain unclear. This study’s objectives were to identify the perinatal and neonatal factors related to LCC and to estimate the factors related to the center variation in the incidence of LCC.

**Methods:**

Extremely preterm infants born between 2008 and 2012 who were registered in the database of the Neonatal Research Network, Japan were retrospectively analyzed. LCC was defined as a clinical diagnosis of LCC and the administration of steroids. We first identified the factors that were significantly related to LCC. We then examined the cause of the center variation in the incidence of LCC, using the standardized incidence ratios (SIRs) of LCC and individual factors.

**Results:**

The factors significantly associated with LCC included low gestational age (odds ratio [OR]: 1.13), small for date (OR: 1.43), male sex (OR: 1.26), antenatal steroid use (OR: 1.19), respiratory distress syndrome (OR: 1.25), chronic lung disease at 36 weeks (OR: 1.16), periventricular leukomalacia (PVL) (OR: 2.57), necrotizing enterocolitis (OR: 0.59), retinopathy of prematurity (ROP) (OR: 1.73), high-frequency oscillating ventilation (HFOV) use (OR: 1.31), parenteral nutrition (OR: 1.38), and red blood cell (RBC) transfusion (OR: 1.94). The SIR of LCC ranged from 0.05 to 2.94, and was positively correlated with SIRs of PVL, ROP, HFOV use and RBC transfusion.

**Conclusion:**

PVL, ROP, HFOV use and RBC transfusion were found to be correlated with the center variation in the incidence of LCC.

## Introduction

Prematurity of adrenal function is recognized as transient adrenocortical insufficiency of prematurity (TAP) or glucocorticoid responsive hypotension in preterm infants [1–3]. In Japan, late-onset circulatory collapse (LCC) was recognized in 2000, and tentative diagnostic criteria were established. In the established criteria, LCC is defined as acute-onset hypotension or oliguria occurring after the transitional period. The incidence of LCC in very low birth weight (VLBW) infants and extremely low birth weight infants is 6.3%, and 11.6%, respectively [4]. Prematurity of adrenal function is an important cause of LCC, and certain drugs, including levothyroxine, may be related to the onset of LCC; however, the etiology remains unclear [5]. The reported incidence of LCC varies among Japanese perinatal centers; however, the cause of such center variation remains unclear [6].

We hypothesized that perinatal and neonatal factors may be related to LCC in preterm infants and these factors could affect center variation in the incidence of LCC. This study attempted to identify the perinatal and neonatal factors associated with LCC in extremely preterm (EP) infants, and to estimate the factors related to center variation in the incidence of LCC in Japan using the nationwide database of the Neonatal Research Network, Japan (NRNJ).

## Materials and methods

### Study design

This was a retrospective case-control study, using a cohort drawn from the NRNJ database containing VLBW infants born between 2003 and 2012, who were treated at 202 participating Japanese perinatal centers. The NRNJ database, which was established in 2003, included perinatal information, neonatal morbidities, interventions and the prognosis of VLBW infants born in Japan. The database was established with the aim of improving the efficacy of searched-based analyses in order to improve the prognosis of VLBW infants. Our study was one of the exploratory studies that used the NRNJ database.

The diagnostic criteria for LCC were: 1) LCC occurred outside the transitional period; 2) a stable period was observed before LCC onset; 3) the absence of any apparent causes prior to LCC onset; 4) the presence of sudden-onset hypotension and/or oliguria; and 5) the hypotension and/or oliguria was resistant to intravenous volume expander and inotropes [4]. In the NRNJ database, LCC was defined as a clinical diagnosis based on the fulfillment of the diagnostic criteria and the administration of steroids. The diagnosis of LCC and decision to administer steroids were made by the physician at each center.

### Study subjects

**Fig 1:**
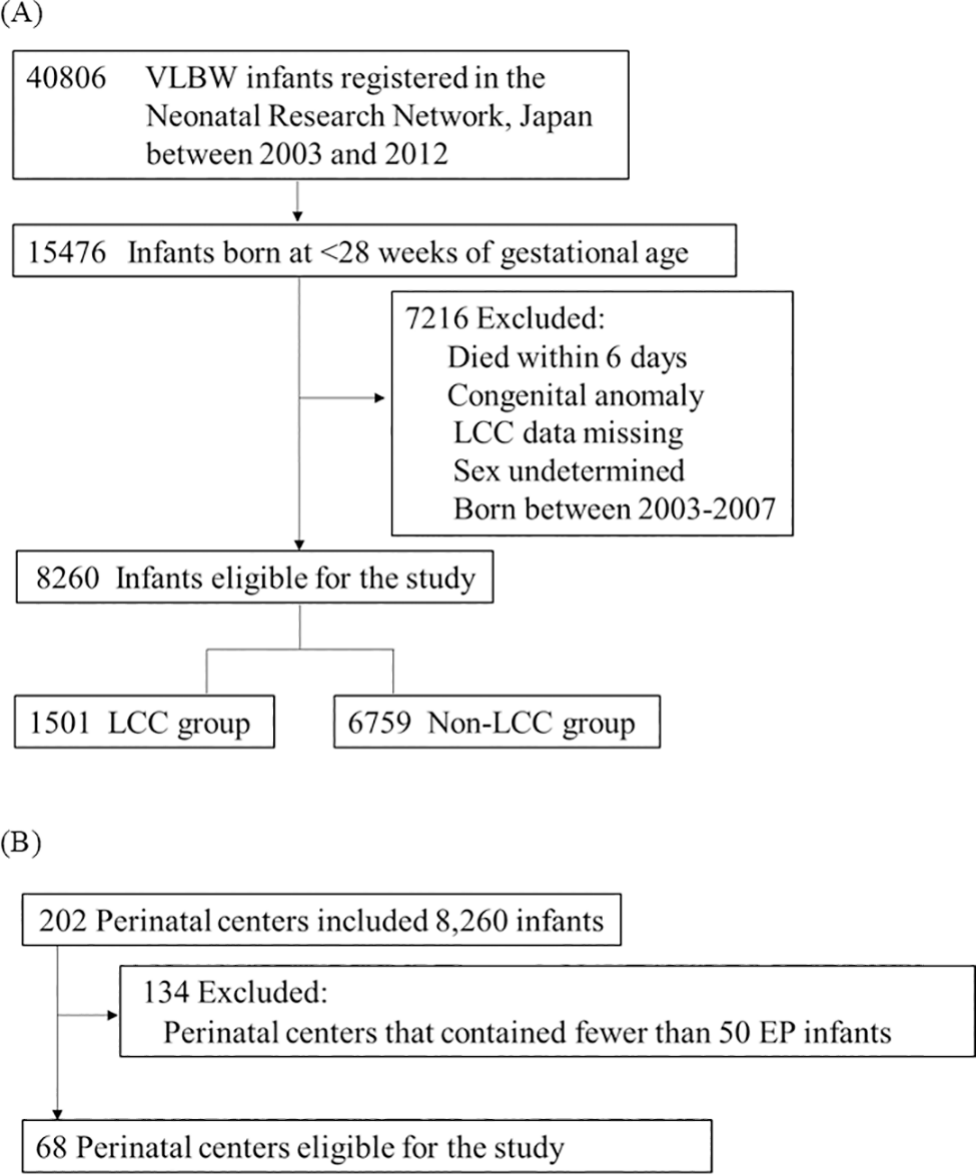
Study subjects. (A) Study subjects for the perinatal and neonatal factors related to LCC. (B) Study subjects for the center variation in the incidence of LCC. VLBW, very low birth weight; LCC, late-onset circulatory collapse; EP, extremely preterm.

VLBW infants (40,806) born between 2003 and 2012 were registered in the NRNJ database (Fig 1(A)). EP infants born between 2008 and 2012 were included in this study because the definition of LCC has been widely recognized in Japanese perinatal centers since 2008. Infants who died within 6 days were excluded according to the definition of LCC. Infants with congenital anomalies, whose sex was undetermined, or whose records were missing data on LCC were also excluded. Consequently, 8,260 EP infants who were born at 22–27 weeks of gestation in 2008–2012 were included in the base study population. In addition, we selected infants from 68 perinatal centers that contained more than 50 EP infants per center during the study period in order to examine the factors related to center variation in incidence of LCC (Fig 1(B)). This study was approved by the Central Internal Review Board of Tokyo Women’s Medical University, where a secretariat of the Neonatal Research Network, Japan (NRNJ) is located; all data were collected and stored anonymously. The institutions enrolled in the study of the NRNJ are described in the Acknowledgements. Jichi Medical University had the approval for participation in the study. All data were fully anonymized before they were accessed by any of the authors.

### Perinatal and neonatal factors related to LCC

We examined 10 perinatal factors that affect the fetal and neonatal adrenocortical function. The gestational age (GA) was determined by ultrasonography, and the mother’s last day of menstruation. Infants whose birth weight and height were below the 10th percentile of the normal curve at each GA were classified as small for date (SFD). Multiple births were determined based on the number of fetuses at delivery. Pregnancy-induced hypertension was defined as maternal hypertension that emerged between 20 weeks of pregnancy and 12 weeks postpartum. Clinical chorioamnionitis was diagnosed based on clinical symptoms, including maternal fever, tender uterus, and an elevated white blood cell count. A non-reassuring fetal status was defined based on a non-reassuring fetal heart rate tracing. Antenatal steroid (ANS) use was defined as the maternal administration of steroids in order to promote fetal lung maturity. Regarding neonatal factors, we examined 10 morbidities and 5 interventions that associated with the prognosis of EP infants. Interventions that were strongly correlated with specific morbidities, such as the use of surfactant which was associated with respiratory distress syndrome (RDS), were avoided. RDS was diagnosed based on the clinical course, the radiographic findings and a microbubble test. Persistent pulmonary hypertension of the newborn was diagnosed based on cardiac sonography showing right-to-left shunt and clinical symptoms. Chronic lung disease (CLD) was defined as needing oxygen for more than 28 days after birth, without congenital malformation. CLD at 36 weeks was defined as severe CLD requiring oxygen at 36 weeks of postmenstrual age. Patent ductus arteriosus (PDA) was defined based on the presence of circulatory failure. To limit the objectives to severe PDA cases, infants requiring ligation were defined as PDA in this study. Intraventricular hemorrhage was defined as grade Ⅲ/Ⅳ cases, according to the classifications of Papile et al. [7]. Cystic periventricular leukomalacia (PVL) was diagnosed based on head sonography or brain MRI. Hypoxic-ischemic encephalopathy was defined based on abnormal findings on head findings on sonography, CT or brain MRI. Sepsis was defined based on the presence of blood culture-positive septicemia. Necrotizing enterocolitis (NEC) was defined as stage Ⅱ/Ⅲ cases, according to the classifications of Bell et al. [8]. Retinopathy of prematurity (ROP) was defined based on the need for ophthalmologic treatment. High-frequency oscillating ventilation (HFOV) use was defined as the use of HFOV during hospitalization in the NICU. Parenteral nutrition (PN) was defined as the use of intravenous hyperalimentation, including amino-acid transfusion. Red blood cell (RBC) transfusion was defined as the performance of RBC transfusion except for cord transfusion during hospitalization in the NICU.

### The standardized incidence ratio of LCC and each perinatal and neonatal factor in the participating centers

The neonatal morbidities and interventions were greatly affected by prematurity, which was indicated by the GA. The standardized incidence ratio (SIR) represents the incidence ratio standardized for every 2 weeks of gestation. In order to equalize distribution of the subjects’ GA among centers, we calculated the SIR of LCC and each perinatal and neonatal factor in the participating centers.

### Statistical analysis

The JMP software program (version 9, SAS Institute Inc.) was used for the statistical analyses. Data are shown as the median and inter-quartile range (IQR) for continuous data, and the percentage for binary data. We compared the perinatal and neonatal factors between the LCC and non-LCC groups by calculating the odds ratio (OR) and 95% confidence intervals (CIs) for each factor using univariate logistic regression analyses. We identified the perinatal and neonatal factors related to LCC by multivariate logistic regression analyses after adjusting for all perinatal and neonatal factors as covariates. Finally, we examined the relationships between the SIRs of LCC and the perinatal and neonatal factors in each center, using linear regression analyses. P values of <0.05 were considered to indicate statistical significance.

## Results

### 1. Comparison of the perinatal characteristics between the LCC and non-LCC groups

The LCC and non-LCC groups included 1,501 and 6,759 cases, respectively. The incidence of LCC in EP infants was 18.1%. According to univariate analyses, the GA and BW were significantly lower and the proportions of SFD, male sex, and ANS use were significantly higher in the LCC group than in the non-LCC group (Table 1). After adjusting for the covariates, multivariate analyses revealed low GA, SFD, male sex, and ANS use to be significantly associated with an increased risk of LCC.

**Table 1:** The perinatal characteristics in the LCC and non-LCC groups and the odds ratios for LCC.

Perinatal factors	LCC (n = 1501)	Non-LCC (n = 6759)	Odds Ratio(95% CI)	
			Unadjusted	Adjusted^†^
GA, week, median (IQR)	25.2 (24.1–26.5)	26.0 (24.7–27.1)	1.27 (1.22–1.31)	1.13 (1.08–1.20)
BW,×100 grams, median (IQR)	6.78 (5.7–8.3)	7.70 (6.3–9.2)	1.22 (1.18–1.25)	–
Small for date, n/N (%)	234/1482 (15.7)	811/6683 (12.1)	1.35 (1.15–1.58)	1.43 (1.16–1.75)
Male sex, n/N (%)	857/1501 (57.1)	3580/6759 (52.9)	1.18 (1.05–1.32)	1.26 (1.11–1.43)
Multiple births, n/N (%)	249/1501 (16.5)	1182/6759 (17.4)	0.93 (0.80–1.08)	0.89 (0.75–1.06)
PIH, n/N (%)	201/1494 (13.4)	789/6737 (11.7)	1.17 (0.99–1.38)	1.01 (0.81–1.26)
Clinical CAM, n/N (%)	462/1435 (32.2)	1903/6430 (29.6)	1.12 (0.99–1.27)	1.10 (0.95–1.27)
NRFS, n/N (%)	352/1451 (24.2)	1460/6496 (22.4)	1.10 (0.96–1.26)	0.99 (0.85–1.15)
Antenatal steroid use, n/N (%)	867/1481 (58.5)	3605/6650 (54.2)	1.19 (1.06–1.33)	1.19 (1.04–1.35)
Cesarean delivery, n/N (%)	1103/1495 (73.7)	4930/6739 (73.1)	1.03 (0.90–1.17)	1.04 (0.89–1.22)

Note. LCC, late-onset circulatory collapse; CI, confidence interval; IQR, interquartile range; GA, gestational age; BW, birth weight; PIH, pregnancy-induced hypertension; CAM, chorioamnionitis; NRFS, non-reassuring fetal status.

^†^Adjusted for GA, small for date, male sex, multiple birth, PIH, clinical CAM, NRFS, antenatal steroid use, cesarean delivery, respiratory distress syndrome, persistent pulmonary hypertension of the newborn, chronic lung disease at 36 weeks, patent ductus arteriosus, intraventricular hemorrhage, periventricular leukomalacia, hypoxic-ischemic encephalopathy, sepsis, necrotizing enterocolitis, retinopathy of prematurity, high-frequency oscillating ventilation use, parenteral nutrition, erythropoietin and red blood cell transfusion.

### 2. Comparison of the neonatal morbidities and interventions in the LCC and non-LCC groups

The mortality rate at discharge was 7.4%, and did not differ between the LCC and non-LCC groups to a statistically significant extent. The causes of death included sepsis, NEC, chronic lung disease, IVH and congenital anomaly (in descending order). According to univariate analyses, the proportions of all neonatal morbidities, except for IVH and NEC, were significantly higher in the LCC group than in the non-LCC group (Table 2). The proportions of all neonatal interventions were also significantly higher in the LCC group than in the non-LCC group. After adjusting for the covariates, multivariate analyses revealed RDS, CLD at 36 weeks, PVL, ROP, HFOV use, PN, and RBC transfusion to be significantly associated with an increased risk of LCC and that NEC was significantly associated with a decreased risk of LCC.

**Table 2:** Neonatal morbidities and interventions in the LCC and non-LCC groups and the odds ratios for LCC.

Neonatal factors	LCC (n = 1501)	Non-LCC (n = 6759)	Odds Ratio (95% CI)	
			Unadjusted	Adjusted^†^
RDS, n/N (%)	1257/1499 (83.8)	5161/6747 (76.4)	1.59 (1.37–1.85)	1.25 (1.05–1.49)
PPHN, n/N (%)	165/1496 (11.0)	515/6732 (7.6)	1.49 (1.24–1.79)	1.05 (0.83–1.30)
CLD at 36 weeks, n/N (%)	761/1458 (52.1)	2480/6508 (38.1)	1.77 (1.58–1.98)	1.16 (1.01–1.32)
PDA, n/N (%)	263/1495 (17.5)	945/6739 (14.0)	1.30 (1.12–1.51)	0.90 (0.75–1.07)
IVH, n/N (%)	136/1498 (9.0)	549/6717 (8.1)	1.12 (0.91–1.36)	0.84 (0.65–1.06)
PVL, n/N (%)	119/1498 (7.9)	210/6723 (3.1)	2.67 (2.11–3.36)	2.57 (1.95–3.36)
HIE, n/N (%)	29/1497 (1.9)	78/6725 (1.1)	1.68 (1.07–2.55)	1.01 (0.57–1.73)
Sepsis, n/N (%)	294/1498 (19.6)	949/6733 (14.0)	1.48 (1.28–1.71)	1.14 (0.96–1.35)
NEC, n/N (%)	52/1499 (3.4)	244/6746 (3.6)	0.95 (0.69–1.28)	0.59 (0.40–0.85)
ROP, n/N (%)	713/1453 (49.0)	1846/6426 (28.7)	2.39 (2.12–2.68)	1.73 (1.51–1.97)
HFOV use, n/N (%)	1044/1480 (70.5)	3515/6612 (53.1)	2.10 (1.86–2.38)	1.31 (1.14–1.52)
Antibiotics, n/N (%)	1358/1494 (90.9)	5766/6685 (86.2)	1.59 (1.32–1.93)	1.02 (0.82–1.29)
Parenteral nutrition, n/N (%)	1352/1496 (90.3)	5588/6741 (82.9)	1.93 (1.61–2.33)	1.38 (1.12–1.71)
Erythropoietin, n/N (%)	1320/1487 (88.7)	5582/6710 (83.1)	1.59 (1.34–1.90)	1.20 (0.98–1.49)
RBC transfusion, n/N (%)	1224/1497 (81.7)	4020/6709 (59.9)	2.99 (2.61–3.45)	1.94 (1.64–2.29)
Mortality at discharge, n/N (%)	101/1501 (6.7)	517/6759 (7.6)	0.87 (0.69–1.08)	–

Note. LCC, late-onset circulatory collapse; CI, confidence interval; RDS, respiratory distress syndrome; PPHN, persistent pulmonary hypertension of the newborn; CLD, chronic lung disease; PDA, patent ductus arteriosus; IVH, intraventricular hemorrhage; PVL, periventricular leukomalacia; HIE, hypoxic-ischemic encephalopathy; NEC, necrotizing enterocolitis; ROP, retinopathy of prematurity; HFOV, High- frequency oscillating ventilation; RBC, red blood cell.

^†^Adjusted for gestational age, small for date, male sex, multiple birth, pregnancy-induced hypertension, clinical chorioamnionitis, non-reassuring fetal status, antenatal steroid use, cesarean delivery, RDS, PPHN, CLD at 36 weeks, PDA, IVH, PVL, HIE, sepsis, NEC, ROP, HFOV use, parenteral nutrition, erythropoietin and RBC transfusion.

### 3. The relationship between the SIR of LCC and the SIRs of perinatal and neonatal factors in each perinatal center

**Fig 2:**
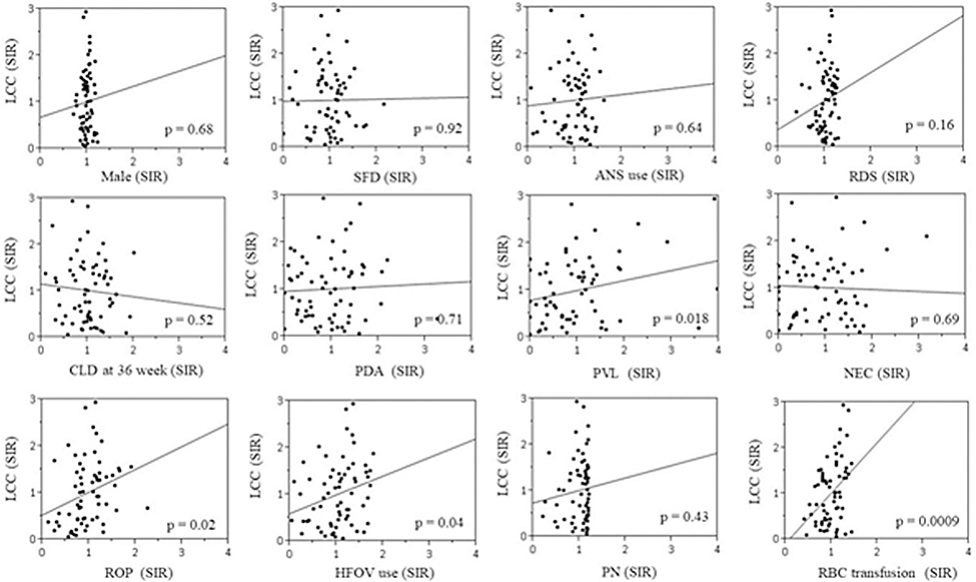
The relationship between the SIRs of LCC and perinatal and neonatal factors in each center. SIR, standardized incidence ratio; SFD, small for date; ANS, antenatal steroid use; RDS, respiratory distress syndrome; CLD, chronic lung disease; PDA, patent ductus arteriosus; PVL, periventricular leukomalacia; NEC, necrotizing enterocolitis, ROP, retinopathy of prematurity; HFOV, high- frequency oscillating ventilation; PN, parenteral nutrition; RBC, red blood cell.

The median number of subjects in each center was 85 (IQR 70–123). Two centers were excluded because their SIRs of LCC (3.71 and 3.19) were considered to be outlying values. The SIR of LCC ranged from 0.05 to 2.94 among 66 perinatal centers. The SIR of LCC was not significantly correlated with the SIRs of any perinatal factors, including SFD, male sex, and ANS use (Fig 2). Conversely, the SIR of LCC in each center showed significant positive correlations with the SIRs of PVL, ROP, HFOV use and RBC transfusion in the corresponding center.

## Discussion

We examined the perinatal and neonatal factors associated with LCC in EP infants using the NRNJ database. GA, SFD, male sex, ANS use, RDS, CLD at 36 weeks, PVL, NEC, ROP, HFOV use, PN and RBC transfusion significantly correlated with LCC. The center variation for LCC remained after standardizing the incidence at every 2 weeks of gestation. The SIR of LCC significantly correlated with the SIRs of PVL, ROP, HFOV use and RBC transfusion.

The incidence of LCC in EP infants was approximately 18% in this study. The perinatal factors significantly associated with LCC included GA, male sex, SFD and ANS use. Previous studies showed that GA was inversely related to the serum cortisol level in the early postnatal period [9–11]. Furthermore, an adrenocorticotropic hormone (ACTH) test showed that the ability to synthesize cortisol was limited in infants of <30 weeks of gestational age [12]. Their immaturity of the hypothalamic-pituitary-adrenal (HPA)-axis and enzymes in the adrenal cortex may lead to adrenocortical insufficiency in infants born at a lower GA. SFD infants also showed higher cortisol levels in the early postnatal period, and blunted HPA-axis responses to stressors [11, 13]. The conditions of SFD infants are similar to those of lower GA infants. ANS use, which suppresses the neonatal adrenal function transiently in the early period after birth, is known to be a cause of TAP [1, 14]. LCC partially overlapped with TAP; thus our results correlated with previous reports.

The neonatal factors significantly related to LCC were RDS, CLD at 36 weeks, PVL, NEC, ROP, HFOV use, PN and RBC transfusion. RDS, CLD at 36 weeks and HFOV use reflected severe respiratory dysfunction. Ng, et al. reported that very low birth weight infants who required mechanical ventilation support showed a weak HPA-axis response on CRH tests at 7 days of age [15]. Severe respiratory dysfunction was thought to be related to the weak response of the HPA-axis and thus, might be the cause of the significant correlation with LCC. With regard to HFOV use, a high mean airway pressure might reduce the cardiac output and thereby cause hypovolemia, leading to circulatory collapse [4].

Regarding LCC onset, most EP infants develop LCC around 25–30 weeks of gestation because LCC generally occurs around third postnatal week [16, 17]. The periventricular avascular area is still present at that time, and the ischemic area due to LCC might result in PVL. Kobayashi, et al. reported LCC to be a risk factor for late-onset cystic PVL, which correlates with our results [18]. The onset of ROP generally occurs at around 30 weeks of gestation and peaks at 36–38 weeks of gestation [19]. Considering the timing of the onset, LCC is thought to mostly occur before ROP. The circulatory fluctuation and lung edema due to LCC increase the supply of oxygen and thus worsen ROP. Contrary to our expectations, NEC was related to a significant decrease in the risk of LCC. This might explain the high mortality rate in NEC patients in the neonatal period. Infants suffering from severe NEC might die before the onset of LCC. Most of these cases were included in the non-LCC group, which may be why NEC was negatively correlated with LCC. Because our study population was sufficiently large, we also calculated the OR and 99% CI for each factor using univariate and multivariate logistic regression analyses (S1 Table). The results did not differ from the results shown in Tables 1 and 2, with the exception of the significance of CLD at 36 weeks in the multivariate logistic regression analysis.

To investigate the center variation in the incidence of LCC, we standardized the incidence ratio of LCC for every 2 weeks of gestation, to equalize subjects’ background difference among centers. To standardize the incidence ratio of LCC for every week of gestation, SFD, and a male sex is desirable; however, the number of subjects in each center was insufficient. The center variation in the incidence of LCC remained after standardization for every 2 weeks of gestation. Furthermore, the SIR of LCC was not significantly correlated with the SIRs of any perinatal factors. We thus concluded that the center variation in the incidence of LCC was not based on differences in the subjects’ perinatal background among centers. The SIR of LCC significantly correlated with the SIRs of PVL, ROP, HFOV use and RBC transfusion in the corresponding centers. The decision of HFOV use was made by the physician at each center, depending on each center’s respiratory management protocol. Thus it might be one of the reasons for the center variation in the incidence of LCC.

The strength of our study is considered to be that large number of the EP infants who developed LCC after the definition of the diagnostic criteria were included and compared with non-LCC infants using the NRNJ database. However, this study is associated with some limitations. First, the timelines of the individual neonatal morbidities and interventions were unknown because our study was retrospective and due to the nature of the database. We therefore could not examine causal associations among the factors. Second, the database did not include a number of factors suspected to cause LCC, including thyroid hormone replacement therapy [5].

In conclusion, GA, SFD, male sex, ANS use, RDS, CLD at 36 weeks, PVL, NEC, ROP, HFOV use, PN, and RBC transfusion were significantly correlated with LCC in EP infants who were registered in the NRNJ database. PVL, ROP, HFOV use and RBC transfusion were found to correlate with the center variation in the incidence of LCC. Prospective studies should be performed to clarify the pathophysiological relationship between these factors and LCC.

## Supporting information

S1 Table The perinatal and neonatal factors in the LCC and non-LCC groups and the odds ratios for LCC. Note. LCC, late-onset circulatory collapse; CI, confidence interval; IQR, interquartile range; GA, gestational age; BW, birth weight; PIH, pregnancy-induced hypertension; CAM, chorioamnionitis; NRFS, non-reassuring fetal status; RDS, respiratory distress syndrome; PPHN, persistent pulmonary hypertension of the newborn; CLD, chronic lung disease; PDA, patent ductus arteriosus; IVH, intraventricular hemorrhage; PVL, periventricular leukomalacia; HIE, hypoxic-ischemic encephalopathy; NEC, necrotizing enterocolitis; ROP, retinopathy of prematurity; HFOV, high- frequency oscillating ventilation; RBC, red blood cell.
^†^Adjusted for GA, small for date, male sex, multiple birth, PIH, clinical CAM, NRFS, antenatal steroid use, cesarean delivery, RDS, PPHN, CLD at 36 weeks, PDA, IVH, PVL, HIE, sepsis, NEC, ROP, HFOV use, parenteral nutrition, erythropoietin and RBC transfusion.
(DOCX)
