# Supplementary material for: Neonatal factors related to center variation in the incidence of late-onset circulatory collapse in extremely preterm infants
Source: PLoS One. 2018 Jun 12;13(6):e0198518. doi: 10.1371/journal.pone.0198518 (PMC5997318; doi:10.1371/journal.pone.0198518)
Supplement: S1 Table — Note. LCC, late-onset circulatory collapse; CI, confidence interval; IQR, interquartile range; GA, gestational age; BW, birth weight; PIH, pregnancy-induced hypertension; CAM, chorioamnionitis; NRFS, non-reassuring fetal status; RDS, respiratory distress syndrome; PPHN, persistent pulmonary hypertension of the newborn; CLD, chronic lung disease; PDA, patent ductus arteriosus; IVH, intraventricular hemorrhage; PVL, periventricular leukomalacia; HIE, hypoxic-ischemic encephalopathy; NEC, necrotizing enterocolitis; ROP, retinopathy of prematurity; HFOV, high- frequency oscillating ventilation; RBC, red blood cell. †Adjusted for GA, small for date, male sex, multiple birth, PIH, clinical CAM, NRFS, antenatal steroid use, cesarean delivery, RDS, PPHN, CLD at 36 weeks, PDA, IVH, PVL, HIE, sepsis, NEC, ROP, HFOV use, parenteral nutrition, erythropoietin and RBC transfusion. (DOCX) [file pone.0198518.s001.docx]

**S1 Table.**

| Factors | LCC ( n=1501) | Non-LCC ( n=6759) | Odds Ratio (99% CI) | |
| --- | --- | --- | --- | --- |
|  |  |  | Unadjusted | Adjusted^†^ |
| GA, week, median (IQR) | 25.2 (24.1-26.5) | 26.0 (24.7-27.1) | 1.27 (1.20-1.33) | 1.13 (1.06-1.21) |
| BW,×100 grams, median (IQR) | 6.78 (5.7-8.3) | 7.70 (6.3-9.2) | 1.22 (1.17-1.27) | － |
| Small for date, n/N (%) | 234/1482 (15.7) | 811/6683 (12.1) | 1.35 (1.10-1.66) | 1.43 (1.09-1.86) |
| Male sex, n/N (%) | 857/1501 (57.1) | 3580/6759 (52.9) | 1.18 (1.01-1.37) | 1.26 (1.06-1.49) |
| Multiple births, n/N (%) | 249/1501 (16.5) | 1182/6759 (17.4) | 0.93 (0.76-1.13) | 0.89 (0.70-1.12) |
| PIH, n/N (%) | 201/1494 (13.4) | 789/6737 (11.7) | 1.17 (0.93-1.45) | 1.01 (0.76-1.35) |
| Clinical CAM, n/N (%) | 462/1435 (32.2) | 1903/6430 (29.6) | 1.12 (0.95-1.32) | 1.10 (0.91-1.33) |
| NRFS, n/N (%) | 352/1451 (24.2) | 1460/6496 (22.4) | 1.10 (0.92-1.31) | 0.99 (0.81-1.21) |
| Antenatal steroid use, n/N (%) | 867/1481 (58.5) | 3605/6650 (54.2) | 1.19 (1.02-1.38) | 1.19 (1.00-1.41) |
| Cesarean delivery, n/N (%) | 1103/1495 (73.7) | 4930/6739 (73.1) | 1.03 (0.87-1.22) | 1.04 (0.85-1.28) |
| RDS, n/N (%) | 1257/1499 (83.8) | 5161/6747 (76.4) | 1.59 (1.31-1.94) | 1.25 (1.00-1.57) |
| PPHN, n/N (%) | 165/1496 (11.0) | 515/6732 (7.6) | 1.49 (1.16-1.90) | 1.05 (0.78-1.39) |
| CLD at 36 weeks, n/N (%) | 761/1458 (52.1) | 2480/6508 (38.1) | 1.77 (1.52-2.06) | 1.16 (0.97-1.38) |
| PDA, n/N (%) | 263/1495 (17.5) | 945/6739 (14.0) | 1.30 (1.07-1.59) | 0.90 (0.71-1.13) |
| IVH, n/N (%) | 136/1498 (9.0) | 549/6717 (8.1) | 1.12 (0.86-1.44) | 0.84 (0.60-1.14) |
| PVL, n/N (%) | 119/1498 (7.9) | 210/6723 (3.1) | 2.67 (1.96-3.62) | 2.57 (1.79-3.65) |
| HIE, n/N (%) | 29/1497 (1.9) | 78/6725 (1.1) | 1.68 (0.93-2.90) | 1.01 (0.47-2.04) |
| Sepsis, n/N (%) | 294/1498 (19.6) | 949/6733 (14.0) | 1.48 (1.22-1.79) | 1.14 (0.91-1.43) |
| NEC, n/N (%) | 52/1499 (3.4) | 244/6746 (3.6) | 0.77 (0.63-1.40) | 0.59 (0.35-0.95) |
| ROP, n/N (%) | 713/1453 (49.0) | 1846/6426 (28.7) | 2.39 (2.05-2.78) | 1.73 (1.45-2.06) |
| HFOV use, n/N (%) | 1044/1480 (70.5) | 3515/6612 (53.1) | 2.10 (1.79-2.47) | 1.32 (1.09-1.59) |
| Antibiotics, n/N (%) | 1358/1494 (90.9) | 5766/6685 (86.2) | 1.59 (1.24-2.05) | 1.03 (0.77-1.39) |
| Parenteral nutrition, n/N (%) | 1352/1496 (90.3) | 5588/6741 (82.9) | 1.93 (1.53-2.47) | 1.38 (1.05-1.83) |
| Erythropoietin, n/N (%) | 1320/1487 (88.7) | 5582/6710 (83.1) | 1.59 (1.27-2.01) | 1.21 (0.92-1.60) |
| RBC transfusion, n/N (%) | 1224/1497 (81.7) | 4020/6709 (59.9) | 2.99 (2.50-3.61) | 1.94 (1.56-2.42) |
